# Adherence of human oral keratinocytes and gingival fibroblasts to nano-structured titanium surfaces

**DOI:** 10.1186/1472-6831-14-75

**Published:** 2014-06-21

**Authors:** Marjan Dorkhan, Tülay Yücel-Lindberg, Jan Hall, Gunnel Svensäter, Julia R Davies

**Affiliations:** 1 Department of Oral Biology, Faculty of Odontology, Malmö University, Malmö SE-205 06, Sweden; 2 Department of Dental Medicine, Division of Periodontology, Karolinska Institutet, Huddinge SE-141 04, Sweden; 3 Nobel Biocare AB, Box 5190, Gothenburg SE- 402 26, Sweden

**Keywords:** Oral keratinocytes, Gingival fibroblasts, Cell attachment, Dental implant, Surface modification, Oral bacteria

## Abstract

**Background:**

A key element for long-term success of dental implants is integration of the implant surface with the surrounding host tissues. Modification of titanium implant surfaces can enhance osteoblast activity but their effects on soft-tissue cells are unclear. Adherence of human keratinocytes and gingival fibroblasts to control commercially pure titanium (CpTi) and two surfaces prepared by anodic oxidation was therefore investigated. Since implant abutments are exposed to a bacteria-rich environment *in vivo*, the effect of oral bacteria on keratinocyte adhesion was also evaluated.

**Methods:**

The surfaces were characterized using scanning electron microscopy (SEM). The number of adhered cells and binding strength, as well as vitality of fibroblasts and keratinocytes were evaluated using confocal scanning laser microscopy after staining with Live/Dead *Bac*light. To evaluate the effect of bacteria on adherence and vitality, keratinocytes were co-cultured with a four-species streptococcal consortium.

**Results:**

SEM analysis showed the two anodically oxidized surfaces to be nano-structured with differing degrees of pore-density. Over 24 hours, both fibroblasts and keratinocytes adhered well to the nano-structured surfaces, although to a somewhat lesser degree than to CpTi (range 42-89% of the levels on CpTi). The strength of keratinocyte adhesion was greater than that of the fibroblasts but no differences in adhesion strength could be observed between the two nano-structured surfaces and the CpTi. The consortium of commensal streptococci markedly reduced keratinocyte adherence on all the surfaces as well as compromising membrane integrity of the adhered cells.

**Conclusion:**

Both the vitality and level of adherence of soft-tissue cells to the nano-structured surfaces was similar to that on CpTi. Co-culture with streptococci reduced the number of keratinocytes on all the surfaces to approximately the same level and caused cell damage, suggesting that commensal bacteria could affect adherence of soft-tissue cells to abutment surfaces *in vivo*.

## Background

Due to their generally good clinical outcomes, dental implants are now a common treatment for replacing lost or missing teeth. The key elements for the long-term success of such implants are the formation of a stable connection between the host bone tissue and the fixture surface (osseointegration) and integration of the transmucosal component, or abutment, with the soft tissues. Previously, much research has concentrated on the optimization of osseointegration but more recently the focus has shifted to include the development of biomaterials that enhance the formation of a peri-implant soft-tissue barrier. In the oral cavity, implant abutments protruding through the mucosa are exposed to a complex environment containing saliva and gingival exudate, as well as microorganisms. The microbial load in the oral cavity is very high, and up to 700 different species have been identified [1]. After placement, the abutment surface rapidly becomes colonized by oral bacteria which can compete with epithelial and connective tissue cells for binding to the surface (for a review see [2]). Typical examples of early colonizers on both hard and soft tissues in the oral cavity are streptococci, including *Streptococcus gordonii*, *Streptococcus oralis*, *Streptococcus mitis* and *Streptococcus sanguinis*, as well as species such as *Actinomyces naeslundii*[3,4]. Adherence of these micro-organisms to the abutment surface can provide binding sites for a new set of colonizers eventually leading to the formation of mature plaque [5,6].

In natural teeth bacterial invasion into the periodontal soft-tissues is limited physically by the gingival mucosa, which forms a seal around the neck of the tooth. The epithelium also acts as a physiological barrier through the release of molecules involved in innate immunity including antimicrobial peptides and cytokines, as well as the initiation of inflammatory responses [7]. In the case of dental implants the natural barrier consisting of junctional epithelium and the periodontal ligament is lacking, thus increasing the importance of a soft-tissue cuff of epithelial cells and fibroblasts tightly attached to the abutment. Studies in dogs have suggested the peri-implant mucosa can form a seal through a cuff of well-keratinized mucosa, analogous to that surrounding natural teeth [8] and layers of epithelial cells have been identified in close association to titanium surfaces implanted into the oral mucosa [9]. In addition, histological studies indicate that epithelial cells can attach to titanium surfaces by means of hemi-desmosomes similar to those found in the internal basal lamina [10]. The arrangement of soft-tissues at the mucosal interface has been shown to be influenced by modification of titanium implant surface topography with oxidisation or acid etching, as well as chemical modification with laminin 5 [11,12]. Recently, the role of nanostructures on titanium implant surfaces in tissue healing has become a major focus of interest. Nanostructures, including nanopores and nanotubues, can be created by anodic oxidation, an electrochemical method in which variables such as voltage, electrolyte concentrations and time can be varied to create different nanotopographies. However, while effects of modifications on the nanometre level on osteoblast activity have been studied extensively [13,14], knowledge of how nanostructures influence peri-implant soft-tissue healing is currently limited. The few *in vivo* studies that have been undertaken in rats [15], dogs [16] or humans [17] (Wennerberg *et al.*) suggest that the use of nanostructured titanium abutment surfaces might improve soft-tissue healing. One *in vitro* study by Zile *et al*. [18] revealed that the density of keratinocytes on nanotubular and nanorough surfaces was increased compared to that on conventional titanium surfaces whereas for fibroblasts, such studies have shown increased cell adherence on nano-structured surfaces prepared by anodic oxidation [19] but decreased adherence to nano-structured titanium coatings on silicone [20]. While these *in vitro* studies have shed light upon possible positive effects of such surfaces, *in vivo*, the situation during the initial stages of healing is much more complex, where cell attachment occurs in the presence of early colonizing oral bacteria such as oral streptococci. Therefore in this study, we have investigated the adherence of human keratinocytes and gingival fibroblasts to nano-structured titanium surfaces and also evaluated the effect of a bacterial consortium containing *Streptococcus gordonii*, *Streptococcus oralis*, *Streptococcus mitis* and *Streptococcus sanguinis* on adhesion of keratinocytes to the surfaces.

## Methods

### Titanium surfaces

Titanium discs (8 mm in diameter and 2 mm in thickness) with three different surfaces were used in this study. Commercially pure titanium discs (CpTi) were used as controls (C) and two anodically-oxidized surfaces (N1 and N2) were used as test surfaces. N1 was prepared by anodic oxidation on commercially pure titanium whereas N2 was prepared by anodic oxidation on titanium alloy (TiAl_6_V_4_). Auger electron spectroscopy analysis revealed the oxide layer on the N1 and N2 surfaces to be greater than 100 nm thick and the mean contact-angles for the control, N1 and N2 surfaces showed no significant differences (51° ± 2, 42° ± 1°, 38° ± 0.3°, respectively) [21]. Profilometry measurements of the mean roughness (Sa) showed that all surfaces had a smooth topography with Sa of 0.18 μm for the control surface, and 0.17 μm and 0.21 μm respectively for the N1 and N2 surfaces [21]. Before use, titanium discs were cleaned by ultrasound treatment in 70% PRO-analys ethanol for 2 × 10 minutes, followed by washing with sterile ultrapure water for a further 2 × 10 minutes.

### Scanning electron microscopy

The surface morphology of the titanium discs was examined using a Zeiss Ultra 55 scanning electron microscope. Images were taken with a secondary electron detector at 8000× magnification using a 10 kV accelerating voltage and an objective lens aperture of 30 μm.

### Human gingival fibroblasts

Human gingival fibroblast cultures were established from gingival biopsies obtained from two healthy subjects (aged 11–13 years) with no clinical signs of periodontal disease. The Swedish Central Ethical Review Board, Stockholm approved the collection of biopsies and the establishment of the fibroblast cell lines (register number 377/98). Minced pieces of gingival tissue were explanted to 25 cm^2^ tissue culture flasks (Falcon) containing 5 ml of DMEM supplemented with penicillin (50 units/ml), streptomycin (50 μg/ml) and 5% fetal calf serum (FCS) (Invitrogen Life Technologies, Scotland, UK). Gingival fibroblasts obtained by trypsinization of the primary cell outgrowth were grown at 37°C with 5% CO_2_ and routinely passaged using 0.025% trypsin in phosphate-buffered saline (PBS) containing 0.02% EDTA at 90% confluence. Human gingival fibroblasts between the 9th and 14th passages were used in this study.

### Human oral keratinocytes

Immortalized normal human oral keratinocytes (OKF6/TERT-2) [22] obtained from Dr James Rheinwald (Brigham and Women’s Hospital, Boston, USA), were seeded into culture dishes in serum-free keratinocyte medium (Gibco) supplemented with 0.2 ng ml^-1^ human recombinant epidermal growth factor, 25 μg bovine pituitary extract ml^-1^ and 0.3 mM CaCl_2_ containing 1 IU penicillin ml^-1^ and 1 μg streptomycin ml^-1^ (DF-K medium) and cultured in 5% CO_2_ in air at 37°C. The medium was changed after 1 day and every 2–3 subsequent days until the cells reached 30-50% confluence. Cells were passaged using 0.05% trypsin-EDTA (Gibco).

### Cell adhesion assay

Titanium discs were placed in 24-well tissue culture dishes (Becton Dickinson), seeded with either keratinocytes or gingival fibroblasts (1 × 10^5^ cells in 1 ml DF-K medium or DMEM supplemented with penicillin (50 units/ml), streptomycin (50 μg/ml) and 5% fetal calf serum, respectively) and incubated in 5% CO_2_ in air at 37°C for 24 hours. This time point was chosen since a sufficient number of cells had adhered in order for the results to be reliable but little cell division had occurred. The discs were gently washed with PBS to remove non-attached cells and stained using Live/Dead *Bac*Light staining kit (Molecular Probes). Adherent cells were visualized using a fluorescence microscope. To evaluate how well the cells were attached to the substratum, a standardised washing procedure was used to remove loosely adhered cells [23]. Discs in culture dishes containing 1 ml PBS were shaken at 100 rpm for 2 × 5 minutes (IKA Vibrax rotary shaker, GMBH & Co., Germany). Cells remaining after this procedure were counted manually after staining with Live/Dead *Bac*Light staining kit and image capture using fluorescence microscopy. The number of cells remaining after the wash was expressed as a ratio of control (number of cells present before washing).

### Bacterial strains

Clinical isolates of *S. gordonii* (HC7), *S. mitis* (BA7), *S. oralis* (89C) and *S. sanguinis* (FC2) were used in this study. *S. gordonii*, *S. mitis* and *S. sanguinis* were obtained from approximal dental plaque, while *S. oralis* was isolated from a peri-implant infection. *S. gordonii* was identified by positive phenotypic tests for *N*-acetyl-glucosaminidase, *N*-acetylgalactosaminidase, α-fucosidase and β-galactosidase while identification of *S. mitis* was based on positive phenotypic tests for *N*-acetyl-galactosaminidase, *N*-acetyl- glucosaminidase, β-galactosidase and sialidase*.* Identification of *S. sanguinis* was based on phenotypic tests negative for sialidase, arbinosidase, L-fucosidase, α-glucosidase and firm adherence to MSA agar and *S. oralis* was identified based on positive phenotypic tests for *N*-acetylglucosaminidase and sialidase and a negative test for D-fucosidase, in addition to sequencing of the *gdh* and *ddl* genes [24]. Bacteria were grown on blood agar in an atmosphere of 5% CO_2_ in air at 37°C. Colonies were transferred to Bacto Todd–Hewitt broth (TH) (Becton Dickinson & Co) and grown overnight in 5% CO_2_ in air at 37°C. The suspensions were transferred to fresh TH broth and incubated at 37°C until the mid-exponential growth phase was reached (A_600_ ≈ 0.5), corresponding to 10^7^ cells/ml. Log phase cultures of each strain were then mixed (1:1:1:1) to give a four-species consortium and centrifuged (4000 *g*) for 10 min. The pellets were then re-suspended in serum-free keratinocyte medium (Gibco) supplemented with 0.2 ng ml^-1^ human recombinant epidermal growth factor, 25 μg bovine pituitary extract ml^-1^ and 0.3 mM CaCl_2_ to give a final concentration of 10^7^ cells/ml.

### Effect of oral bacteria on keratinocyte adherence

To study the effect of oral bacteria on keratinocyte adhesion, titanium discs were seeded with 1 ml of OKF6/Tert 2 cells and these were allowed to attach for 16 hours. After this time, 1 ml of the four-species bacterial consortium containing 10^7^ cells in serum-free keratinocyte medium as described above, was added and the discs then incubated in 5% CO_2_ in air at 37°C for an additional 8 hours. After a total incubation time of 24 hours, the discs were washed gently with serum-free keratinocyte medium to remove non-attached cells and the remaining adherent cells stained with Live/Dead *Bac*Light staining kit (Molecular Probes) prior to visualization in a fluorescence microscope. To evaluate strength of attachment of the bacteria-treated cells to the substratum, the same standardised washing procedure as outlined above was used. Alternatively, titanium discs were seeded with 1 ml of the four-species bacterial consortium containing 10^7^ cells in serum-free keratinocyte medium (5% CO_2_ in air at 37°C) for 2 hours and washed three times with serum-free keratinocyte medium before the addition of 1 × 10^5^ cells in 1 ml serum-free keratinocyte medium for 22 hours.

### Image analysis and statistics

Experiments were carried out three times using independent cell and bacterial cultures. For all experiments, images were taken at the same twenty standardized points on the surfaces, avoiding the centre and extreme edges of the discs. The results for the test surfaces were compared to control using ANOVA, with a Bonferroni post-test and p-values below 0.05 were considered significant.

## Results

### Surface morphology

**Figure 1:**
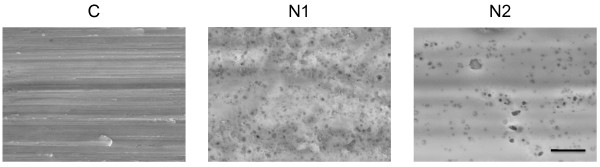
**SEM images of CpTi and anodically oxidized surfaces.** Representative images of the control (CpTi) (C), and anodically oxidized surfaces (N1 and N2). The scale bar represents 1 μm.

The surface SEM micrographs of the CpTi control and anodically-oxidized surfaces, are shown in Figure 1. The surface of the CpTi control disc had an anisotropic topography similar to that of machined commercial implants. Both the anodically-oxidized surfaces (N1 and N2) had pores in the 50 nm range. On the N1 surface, the pore-density was high and the pores were evenly distributed over the surface whereas on the N2 surfaces, the pores more sparsely distributed and clustered, giving rise to larger pore-free areas.

### Attachment of human gingival fibroblasts to nano-structured titanium surfaces

**Figure 2:**
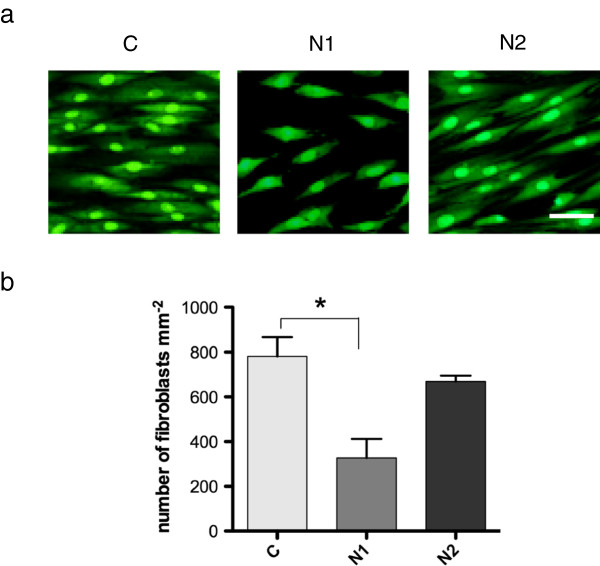
**Adherence of human gingival fibroblasts to CpTi control (C) and nano-structured (N1 and N2) surfaces. (a)** Adhered cells were stained with Live/Dead *Bac*Light and viewed in a fluorescence microscope. The scale bar represents 50 μm. **(b)** Graphs showing mean number of adhered cells ± SEM from three independent experiments. Data were analyzed using ANOVA with a Bonferroni post-test (*p < 0.05).

Adherence of human gingival fibroblasts to the three surfaces, CpTi control, N1 and N2 was investigated. Cells were allowed to adhere for 24 hours, prior to staining with *Bac*light Live/Dead and visualization in a fluorescence microscope. Image analysis revealed the cells to have an elongated morphology characteristic of fibroblasts (Figure 2a). On the CpTi control surface, the cell density was relatively high (Figure 2a), corresponding to approximately 781 ± 87 cells mm^-2^ (Figure 2b). Levels of coverage for the N2 surface were (669 ± 26 cells mm^-2^), corresponding to 85% of control, whereas coverage on the N1 surface (328 ± 84 cells mm^-2^) corresponded to 42% of control (Figure 2a,b). The reduction on the N1 surface was significant at the 5% level compared to control. Thus, the gingival fibroblasts showed a somewhat lower capacity to adhere to the N1 than to the control and N2 surfaces.

An assay, based on the number of cells removed by a standardized washing procedure, was used to determine the adhesion strength of the fibroblasts on the three surfaces. The number of adherent cells remaining after the treatment was quantified and calculated as a percentage of the initial cell count on the same surface. This revealed that on the CpTi control surface, 33 ± 0.7% of the cells remained after washing while for the N1 and N2 surfaces, the corresponding figures were 44 ± 6.8% and 23 ± 2.7% respectively. These data indicate that there was no difference between the strength of adhesion of the gingival fibroblasts to the CpTi control and the nano-structured surfaces.

### Adherence of oral keratinocyes to titanium surfaces

**Figure 3:**
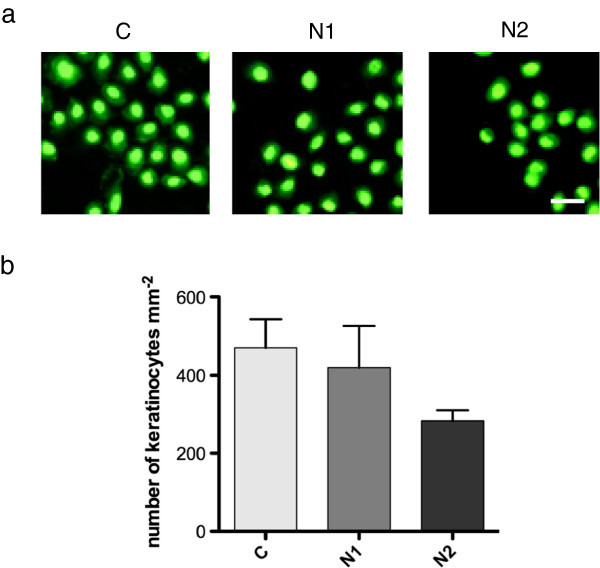
**Adherence of human oral keratinocytes to CpTi control (C) and nano-structured (N1 and N2) surfaces. (a)** Adhered cells were stained with Live/Dead *Bac*Light and viewed in a fluorescence microscope. The scale bar represents 50 μm. **(b)** Graphs showing mean number of adhered cells ± SEM from three independent experiments.

The other soft-tissue cells of importance for integration of dental implants are oral keratinocytes, and therefore the adherence of these cells to the three surfaces was also tested. After 24 hours, the keratinocytes were viable and were found as clusters of cells with a typical polygonal morphology, spread evenly over the surface of the titanium discs (Figure 3a). On the CpTi control surface, the mean number of cells was 470 ± 73 mm^-2^. The mean number of cells on the N1 surface (419 ± 107 mm^-2^) was similar to that on the CpTi whereas that on the N2 surface was somewhat lower (284 ± 27 mm^-2^). These values correspond to 89% and 60%, respectively, of the level on the control surface. None of these differences was significant at the 5% level (Figure 3b). The adhesion strength of the keratinocytes on the surfaces was investigated using the washing assay. Similar proportions of the attached cells remained on all three surfaces after washing, suggesting that the adhesion strength of the keratinocytes was not influenced by the presence of nano-structures (left column, Table 1).

**Table 1:** Keratinocytes on CpTi (C) and nano-structured surfaces (N1 & N2) after a standardized wash, expressed as a percentage of the pre-wash levels for the same surface

**Keratinocytes remaining on surfaces after wash**		
**Surface**	**- Bacteria**	**+ Bacteria**
C	83 ± 5.5%	96 ± 0.3%
N1	75 ± 1.4%	83 ± 3.5%
N2	94 ± 1.5%	87 ± 4.6%

### Influence of oral streptococci on binding of keratinocytes

**Figure 4:**
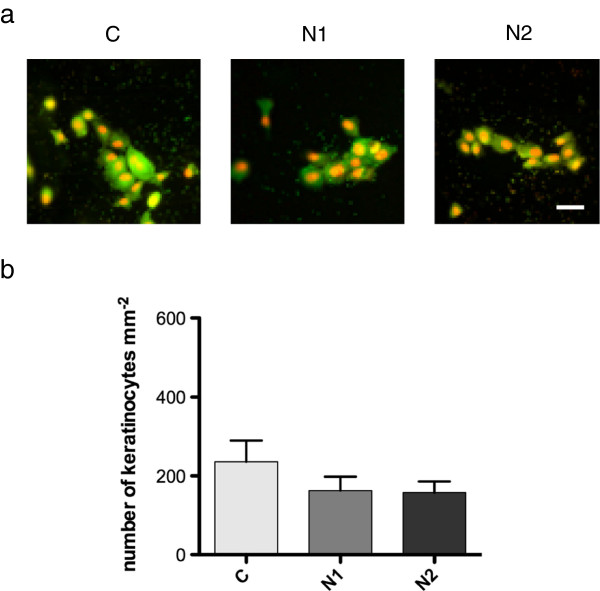
**Adherence of human oral keratinocytes to CpTi control (C) and nano-structured (N1 and N2) surfaces incubated with a consortium of oral streptococci. (a)** Adhered cells were stained with Live/Dead *Bac*Light and viewed in a fluorescence microscope. The scale bar represents 50 μm. **(b)** Graphs showing mean number of adhered cells ± SEM from three independent experiments.

**Figure 5:**
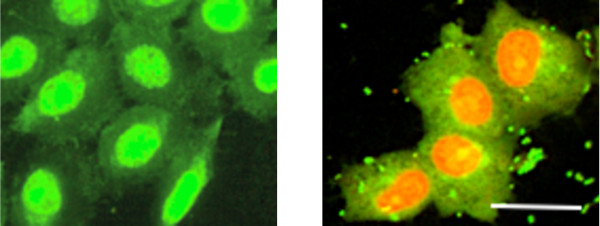
**Effects of a consortium of oral streptococci on keratinocytes.** Human oral keratinocytes were grown on control surfaces (C) in the absence (left panel) or presence (right panel) of a consortium of oral streptococci. The scale bar represents 50 μm.

In the area where the abutment emerges through the oral epithelium, keratinocytes attaching to the abutment surface are exposed to the oral microbiota. Therefore the influence of a consortium of early colonizers on keratinocyte adherence was investigated. The presence of bacteria during the final 8 hours of the adherence period caused a marked reduction in the number of keratinocytes found on all the surfaces compared to the level seen in the absence of bacteria (Figure 3b), although this difference was not significant at the 5% level (Figure 4a,b). Small numbers of bacteria were found as clusters associated with both the titanium surface and the adherent keratinocytes. These data thus suggest that the presence of bacteria either reduced the ability of the keratinocytes to bind to the surfaces or caused detachment of adhered cells. Interestingly, the nucleus of the keratinocytes exposed to the bacteria for 8 hours showed red or orange staining with the *Bac*light Live/Dead stain that was not seen in the unexposed keratinocytes (Figure 5). This indicates that propidium iodide had entered the cells and is indicative of damage to the cell membrane. The presence of the bacteria thus, not only appeared to cause a reduction in the net number of cells adhering to the substrate after 24 hours, but also affected cell viability. To investigate the effect of bacteria on adhesion strength of keratinocytes, the washing assay was also applied to cells previously exposed to the bacteria. After this treatment, no significant decrease in the number of cells present on the surface compared to the pre-wash levels was seen for any of the surfaces (right column, Table 1). These results therefore suggest the bacteria did not affect the strength of adhesion of the keratinocytes to the different surfaces. The adherence of keratinocytes to surfaces already colonized by the consortium of oral streptococci was also investigated. After 2 hours, surface coverage by the consortium was around 50% and 20 hours after their addition, only sporadic adherence of keratinocytes was seen (data not shown). Thus the presence of early colonizing bacteria on the titanium surfaces clearly inhibited subsequent attachment of keratinocytes.

## Discussion

Previously, much work has focussed on the modification of TiO_2_ surfaces to improve osseo-integration and studies have investigated both osteoblast adherence *in vitro*[13,14] and tissue integration *in vivo*[25]. However, there are still significant gaps in our knowledge regarding the adherence and function of soft-tissue cells at the abutment-mucosa interface. In this study, adherence of keratinocytes and fibroblasts to two surfaces; one formed by anodic oxidation on CpTi (N1) and the other, formed by anodic oxidation on a titanium alloy (N2), was compared to that on a control CpTi surface. Both the modified surfaces were nano-structured with pores in the 50 nm range covering the entire surface. However, they differed in pore-density, with more sparsely distributed pores and with larger pore-free areas on the N2 than on the N1 surface. As well as nanoscale modifications, anodic oxidation can cause changes in the bulk material surface, and the TiO_2_ surface layers of N1 and N2 have been demonstrated to contain higher levels of crystalline anatase than the CpTi control [21]. Although cytotoxic effects on bacteria have been observed in the presence of UV radiation [26], little is known about the effects of surface-associated anatase on eukaryotic cells. In this study, however, the anatase-rich N1 and N2 surfaces demonstrated no cytotoxic effects on either fibroblasts or keratinocytes.

The pattern of binding of human gingival fibroblasts was somewhat different to that of keratinocytes. Both fibroblasts and keratinocytes were able to bind to the CpTi control and the nano-structured surfaces. However, while the level of adherence of fibroblasts to the N2 surfaces was similar to that on CpTi control, adherence to the N1 surface was significantly reduced. For keratinocytes, even though the number of cells was somewhat lower on the N2, there were no significant differences in the overall cell density on the nano-structured surfaces compared to that on CpTi control. The data thus show that the presence and distribution of pores had no influence on the adherence of oral keratinocytes whereas for gingival fibroblasts, a high pore-density reduced adherence. This suggests that binding of gingival fibroblasts was affected by the nature of the substrate whereas that of keratinocytes was not influenced to the same extent. In a study by Ma *et al*., the presence of nanotubules with a diameter of around 100 nm reduced fibroblast adherence compared to a polished titanium control [27] while Guida *et al*. showed increased fibroblast adherence to oxidized nano-structured surfaces compared to turned titanium controls [19]. Conclusions regarding the effects of nano-structures on fibroblast function are thus difficult to draw from the current literature. For keratinocytes, data is limited but Zile *et al*. [18] have shown that nano-tubular titanium surfaces promote adherence compared to conventional nano-smooth surfaces. In this study, the number of keratinocytes adhering to the conventional titanium surface was comparable to that seen for the control surfaces in their study, although no increase in cell density on the nano-structured surfaces used here was observed.

The formation of a tight seal between the implant and the surrounding soft-tissues is expected to contribute to the maintenance of healthy tissues around an implant [28,29]. Studies conducted in dogs have revealed that sol–gel derived nanoporous titanium surfaces can induce the development of hemidesmosomal interactions between the oral mucosa and the implant surface [16]. Similarly, in human subjects, contact between the oral mucosa and titanium implants was enhanced in the presence of sol–gel-derived titanium oxide films [17]. Therefore the adhesive strength of the keratinocytes and fibroblasts was investigated using a procedure that removed weakly attached cells. A similar method has been used previously to investigate the adherence of epithelial cells and fibroblasts to titanium [23]. The results of this study revealed no differences in adhesion strength of keratinocytes or fibroblasts to the anodically-oxidized surfaces compared to CpTi control suggesting that the nano-structures did not influence the strength of attachment of either cell type*.* This is in agreement with findings from another *in vitro* study where adhesion strength of fibroblasts was not affected by surface conditions and materials [30]. However fibroblasts were removed to a greater extent from all of the surfaces than the keratinocytes, indicating a lower overall adhesive strength for these cells.

Most *in vitro* studies of the influence of implant surface properties on host cell adherence have been undertaken in bacteria-free environments. However when an abutment surface is placed in the oral cavity, the capacity of soft-tissue cells, particularly keratinocytes, to adhere may be influenced by the presence of microorganisms in the oral environment. The effect of a consortium of early colonising streptococci on adherence and adhesion strength of keratinocytes was therefore investigated. Keratinocyte adherence was largely inhibited when bacteria were already present on the titanium surfaces suggesting that soft-tissue healing could be strongly influenced by the presence of microbial biofilms on the abutment surface. Exposure of attaching keratinocytes to the same bacteria, reduced cell density by around 50% and the integrity of the cell membrane of the remaining cells appeared to be compromised. While the cell damage observed here was somewhat surprising since oral streptococci are normally not considered to be pathogenic, similar results were obtained in *in vitro* studies where osteoblasts were exposed to the skin commensal, *Staphylococcus epidermidis*[31,32]. It is known that commensal streptococci produce a range of metabolic end-products including lactate, acetate, ethanol and formate as well as enzymes (glycosidases and proteases) and bacterial antigens including lipoteichoic acid (LTA) which may be considered as possible mechanisms for the observed cell damage [33,34]. While keratinocytes can respond to LTA through Toll-like receptors [35], the effects of other substances upon keratinocyte function are currently not well understood. No reduction in adhesion strength of keratinocytes, co-cultured with bacteria, to the titanium surfaces could be detected suggesting that the cells were not less strongly attached than their counterparts in sterile conditions.

## Conclusions

A major challenge in dental implant research is to develop surfaces that diminish biofilm formation at the same time as promoting adherence of host cells (keratinocytes, fibroblasts and osteoblasts). Here, only minor differences were seen in the adherence of keratinocytes and fibroblasts to the nano-structured surfaces compared to the CpTi control. In a previous study, adherence of oral streptococci to the N1 and N2 surfaces was significantly lower than to the CpTi control [21] suggesting that these surfaces may be capable of promoting host tissue cell adherence while minimizing bacterial adhesion. Thus, anatase-rich, nano-structured surfaces appear to offer promising properties for enhancement of dental implant abutment integration with soft-tissues however, this process was negatively affected by the presence of commensal oral bacteria.

## Abbreviations

CpTi: Commercially pure titanium; LTA: Lipotechoic acid; SEM: Scanning electron microscopy; UV: Ultraviolet radiation.

## Competing interests

The authors declare that they have no competing interests.

## Authors’ contributions

MD participated in planning and designing the study, performed most of the laboratory work and participated in the data analysis and drafting of the manuscript. GS, JRD, TL and JH participated in study design, data analysis and drafting of the manuscript. All authors have read and approved the final manuscript.

## Pre-publication history

The pre-publication history for this paper can be accessed here:

http://www.biomedcentral.com/1472-6831/14/75/prepub
